# 2456. Methicillin-Resistant *Staphylococcus aureus* Bacteremia 30-Day Mortality During 2020 Compared to 2016-2019: Assessing the Impact of COVID-19

**DOI:** 10.1093/ofid/ofad500.2074

**Published:** 2023-11-27

**Authors:** Holly Biggs, Kelly A Jackson, Joelle Nadle, Susan Petit, Susan M Ray, Ghinwa Dumyati, Marissa Tracy, Tiffanie M Markus, William Schaffner, Isaac See, Isaac See

**Affiliations:** CDC, Atlanta, Georgia; U.S. Centers for Disease Control and Prevention, Atlanta, Georgia; California Emerging Infections Program, Oakland, California; Connecticut Department of Public Health, Hartford, Connecticut; Emory University School of Medicine, Atlanta, Georgia; New York Emerging Infections Program and University of Rochester Medical Center, Rochester, New York; University of Rochester Medical Center, Rochester, New York; Vanderbilt University Medical Center, Nashville, Tennessee; Vanderbilt University Medical Center, Nashville, Tennessee; U.S. Centers for Disease Control and Prevention, Atlanta, Georgia; U.S. Centers for Disease Control and Prevention, Atlanta, Georgia

## Abstract

**Background:**

Methicillin-resistant *Staphylococcus aureus* (MRSA) bacteremia is associated with substantial mortality, but the effect of the COVID-19 pandemic on MRSA mortality has not been fully explored.

**Methods:**

For 2016-2020, MRSA bacteremia cases among adults aged ≥ 18 years were identified through CDC Emerging Infections Program’s active laboratory- and population-based surveillance in 5 states (16 counties). Death within 30 days of initial MRSA blood culture was determined from medical record documentation or linked state vital statistics data. Cases were classified (epi class) as hospital-onset (HO), healthcare-associated community onset (HACO), or community associated (CA) (Figure 1). Thirty-day mortality was calculated by year and epi class and, for 2020, stratified by whether a SARS-CoV-2 test was positive in the 30 days preceding MRSA culture (recent COVID-19). Using multivariate logistic regression to adjust for potential confounders, the effect of recent COVID-19 on 30-day mortality was assessed.

**Figure:**
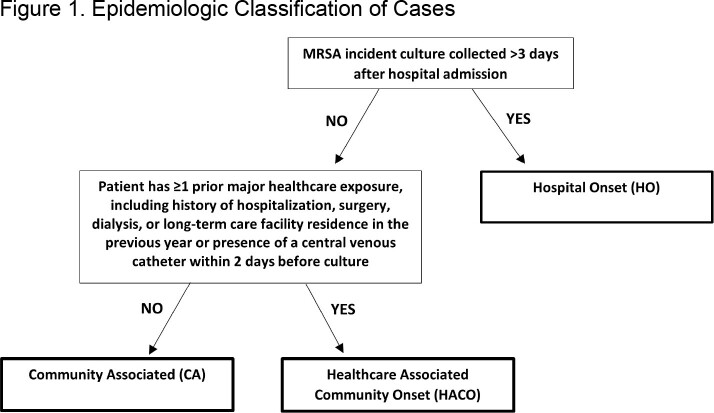

**Results:**

MRSA bacteremia 30-day mortality was 22% (2,113/9,689) during 5 surveillance years. Mortality in 2020 was higher than in 2016-2019 overall (25% vs. 21%, p< .01), for HO cases (43% vs 32%, p< .01), and for CA cases (19% vs. 13%, p< .01). When limited to cases without recent COVID-19, mortality in 2020 was still higher than in 2016-2019 for CA (18%, p< .01) but not HO (35%, p=.39). During 2020, cases with recent COVID-19 comprised 37% of HO deaths and a smaller proportion of HACO (13%) and CA (7%) deaths. Cases with recent COVID-19 had higher 30-day mortality than those without across all epi classes (Figure 2; p≤ .01 for all), with mortality highest among HO cases with recent COVID-19 (77%). After adjusting for other factors, recent COVID-19 remained a significant risk factor for 30-day mortality (adjusted odds ratio: 5.3, 95% CI: 3.6-8.0; Table).

**Figure:**
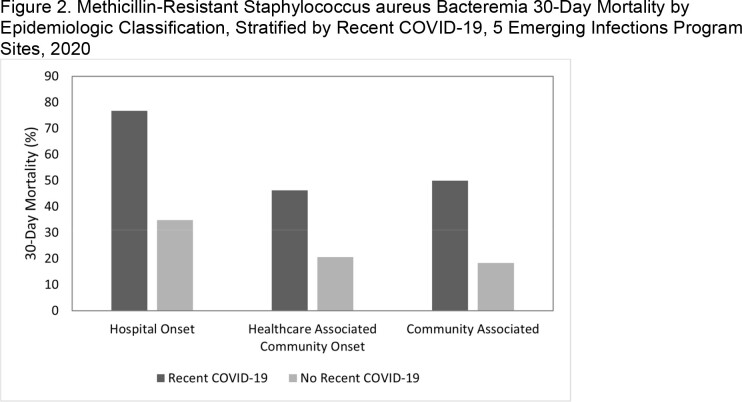

**Table.**
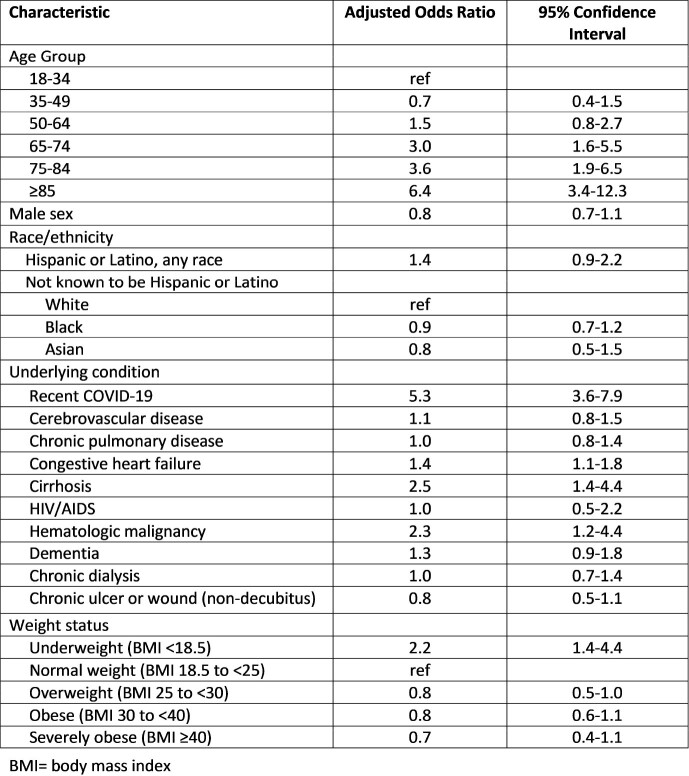
Multivariate Analysis of Factors Associated with Methicillin-Resistant Staphylococcus aureus Bacteremia 30-Day Mortality, 5 Emerging Infections Program Sites, 2020

**Conclusion:**

COVID-19 emerged as a significant independent risk factor for MRSA bacteremia-associated death in 2020, and the very high mortality for HO cases with recent COVID-19 resulted in a higher mortality for HO cases overall. Conversely, the contribution of COVID-19 to the increased mortality for CA cases in 2020 appeared small, and reasons for the increase in mortality for CA cases without recent COVID-19 need additional investigation.

**Disclosures:**

**Ghinwa Dumyati, MD**, Pfizer: Grant/Research Support

